# Study protocol for a modified antenatal care program for pregnant women with a low risk for adverse outcomes—a stepped wedge cluster non-inferiority randomized trial

**DOI:** 10.1186/s12884-022-04406-7

**Published:** 2022-04-08

**Authors:** Valerie Stålberg, Barbro Krevers, Lovisa Lingetun, Therese Eriksson, Ann Josefsson, Caroline Lilliecreutz

**Affiliations:** 1 Department of Obstetrics and Gynecology, Division of Children’s and Women’s Health, Department of Biomedical and Clinical Sciences, Faculty of Medicine and Health Sciences, Linköping University, 581 85 Linköping, Sweden; 2 Department of Health, Medicine and Caring Sciences, Linköping University, Linköping, Sweden

**Keywords:** Antenatal care, Risk assessment, Low risk pregnancy, Maternal outcomes, Neonatal outcomes, Virtual care, Patient satisfaction, Caregiver satisfaction, Health economics, e-health

## Abstract

**Background:**

It is crucial to provide care based on individual needs. Swedish health care is obliged to give care on equal conditions for the entire population. The person with the greatest need should be given the most care, and the health care system should strive to be cost-efficient. Medical and technical advances have been significant during the last decades and the recent Covid-19 pandemic has caused a shift in health care, from in-person visits to virtual visits. The majority of pregnant women with a low risk assessment have an uncomplicated antenatal course without adverse events. These women probably receive excessive and unnecessary antenatal care. This study will investigate if an antenatal care program for healthy pregnant women with a low risk for adverse outcomes could be safely monitored with fewer in-person visits to a midwife, and with some of them replaced by virtual visits.

**Methods:**

This is a non-inferiority trial where a stepped wedge cluster randomized controlled design will be used. Data collection includes register data and questionnaires that concern antenatal, obstetric and neonatal outcomes, patient- and caregiver-reported experiences, healthcare-economy, and implementation aspects. The modified antenatal care (MAC) study is performed in parts of the southeast of Sweden, which has approximately 8200 childbirths annually. At the start of the study, all antenatal care centers included in the study will use the same standard antenatal care (SAC) program. In the MAC program the in-person visits to a midwife will be reduced to four instead of eight, with two additional virtual meetings compared with the SAC program.

**Discussion:**

This presented study protocol is informed by research knowledge. The protocol is expected to provide a good structure for future studies on changed antenatal care programs that introduce virtual visits for healthy pregnant women with a low risk for adverse outcomes, without risking quality, safety, and increased costs.

**Trial registration:**

The study is registered the 21th of April 2021 in the ISRCTN registry with trial ID: ISRCTN14422582, retrospectively registered.

**Supplementary Information:**

The online version contains supplementary material available at 10.1186/s12884-022-04406-7.

## Background

Historically, antenatal care programs were mainly developed to detect preeclampsia and gestational diabetes mellitus. Medical and technical advances in the last decades and new tests to detect complications e.g. the oral glucose tolerance test and screening for the risk of preeclampsia have been included in the antenatal care. There is a lack of evidence on the effectiveness, frequency and timing of the scheduled antenatal care visits. Studies regarding antenatal care programs has looked at different models and concluded that a reduction in the number of antenatal care visits during pregnancy is safe regarding neonatal and obstetric outcomes [1]. Also, no differences have been shown in adverse events when considering women having antenatal visits to an obstetrician, compared to midwives or general practitioners [2]. The clinical content of the antenatal care program has been subject to several evaluations and is still controversial. Most guidelines recommend routine assessment with fundal height, maternal weight, blood pressure measurements, fetal heart auscultation, urine testing for protein and questions about fetal movements. The evidence supporting these practices is variable and in most cases, no association between the assessment and improved maternal or fetal outcomes has been proven [3–8]. In addition, antenatal care has three main psychological functions: to provide information, social support and reassurance for reducing antenatal and postnatal morbidity. Studies have shown that the quality of the visit is crucial for the patient’s satisfaction rather than the number of visits to the midwife [9–11].

The recent Covid-19 pandemic has caused a shift from in-person visits to virtual visits. Peahl et al. conducted a study in 2020 with a redesign of the antenatal care for pregnant women with a low risk, consisting of four in-person visits, one obstetric ultrasound and four virtual visits [12]. E-health has become a promising option for providing healthcare, and early trials using virtual care and remote monitoring have shown high patient satisfaction [13].

The current standard antenatal care (SAC) program was developed by the Swedish national reference group for maternal health care during the early 1990s and has undergone some minor revisions regarding frequency and timing of the scheduled midwife visits during the last decades. At the same time, the medical content has expanded, now including for example screening for anemia, thyroid disease, evaluation for thromboembolic risk and anti-Rhesus immunization during pregnancy.

The SAC program in Sweden is free of charge with a high attendance rate of almost 100%. There is no longer a routine visit to an obstetrician for all pregnant women, but instead the midwife carries out a risk assessment based on national guidelines in early pregnancy. The risk assessment determines if the SAC program is sufficient or if extra visits are needed, i.e., for an ultrasound for fetal growth or an appointment with an obstetrician.

According to the Swedish Health Care Act, publicly funded care is obliged to follow ethical principles for prioritization and resource allocation, namely the human dignity principle, the need-solidarity principles and the cost-effectiveness principle. That means that all people must be treated with respect for their dignity and considered as having equal value, those with the greatest need for health care should be given priority, and health care activities should be organized so that cost-effectiveness is promoted [14]. The majority of pregnant women with a low risk assessment in early pregnancy have an uncomplicated antenatal course without adverse events during pregnancy and childbirth [15]. These women probably receive excessive and unnecessary antenatal care in Sweden today.

The proportion of women with a high risk for complications during pregnancy and childbirth is increasing due to e.g. obesity, advanced age or intercurrent diseases [16, 17]. It is therefore important to individualize the antenatal care visits to a greater extent and make a resource-shift toward those who need more care. That is to ensure that the resources are appropriately and efficiently distributed to the care of those with the greatest need, which is of particular importance in a publicly funded health care system.

Our hypothesis is that the modified antenatal care (MAC) program does not differ in regard to antenatal, obstetric and neonatal outcomes compared to the SAC program. We also hypothesize that the cost of the MAC program is lower than the cost of the SAC program. We also assume that selected implementation strategies will support a high level of adherence and sustainability for the MAC program.

This study protocol will describe the guideline development of a MAC program. The study design and methods aimed to investigate if a reduction in-person visits to a midwife with some of those visits replaced with virtual visits is safe for healthy pregnant women with a low risk for adverse outcomes. The planned study will evaluate a MAC program in comparison to the SAC program for healthy pregnant women with a low risk for adverse outcomes, considering the following aspects: maternal- and neonatal outcomes, patient- and caregiver-reported experiences, healthcare economy, and will also evaluate the MAC program implementation.

## Methods

### Study design

This is a stepped wedge cluster randomized controlled trial comparing two different programs: the standard antenatal care (SAC) program and the modified antenatal care (MAC) program in five antenatal care centers (ACC) where each participating ACC constitutes one cluster (A-E). All visits to both the SAC and MAC take place in an ACC in the South East region of Sweden. At the start of the study, all ACCs will use the same and current SAC program. The healthy pregnant women with a low risk for adverse outcomes, who are attending the current SAC program will constitute the control group. After the start of the MAC program, the corresponding women will constitute the index group.

### Procedures

#### Guideline development

To scrutinize all aspects of antenatal care, we first assembled a research team consisting of senior consultants in maternity and obstetric care, midwives, a health economist, experts in implementation strategies, and in the later stages also statistics experts, hospital administration staff, health communicators, IT consultants and executives. We used systematic literature reviews and PubMed searches to investigate existing evidence in the fields of antenatal care and e-health. After having drawn up the recommended checks during pregnancy, they were divided into two categories: the ones who we assumed would need in-person visits i.e., ultrasounds, laboratory tests and physical examination, and the others that we assumed could be offered remotely through virtual meetings, mainly concerning information and reassurance. These controls were thereafter grouped, based on their recommended timing during pregnancy. With the introduction of the MAC program the in-person visits to a midwife will be reduced to four instead of eight, including the visit with the risk assessment, with two additional virtual meetings. Two obstetric ultrasounds are conducted in both the SAC and MAC, see Table 1. The first meeting in both the SAC and the MAC program is an in-person visit to establish a bond of trust between the pregnant woman and the midwife. That has been shown to be preferred by both patients and caregivers when using blended care, i.e., mixing in-person visits and virtual visits [18, 19]. During this visit a thorough risk assessment is carried out and the midwife determines if the pregnant women has a low or high risk for complications during pregnancy, see Supplement 1. In early pregnancy, before the risk assessment visit, information about recommended life-style changes in pregnancy is given to the pregnant women.

**Table 1:** Main medical content in the standard antenatal care program (SAC) and the modified antenatal care program (MAC)

	**SAC**		**MAC**		
Gestational week	In-person visit	Medical content e.g	In-person visit	Virtual visit	Medical content e.g
6 to 10		Lifestyle recommendations			Lifestyle recommendations
11 to 15	# 1	Risk assessment Gynecological examination Blood pressure Prenatal labs Urine testing for protein	# 1		Risk assessment Gynecological examination Blood pressure Prenatal labs Urine testing for protein
11 to 13 + 6	yes	Obstetrical ultrasound with preeclampsia risk assessment	yes		Obstetrical ultrasound with preeclampsia risk assessment
18 + 0 to 20 + 0	yes	Obstetrical ultrasound	yes		Obstetrical ultrasound
25	# 2	Blood pressure Fetal heart rate with Doppler Symphysis fundus height Plasma-glucose		# 1	Up-dating history
29	# 3	Blood pressure Fetal heart rate with Doppler Symphysis fundus height Plasma-glucose Blood tests Urine testing for protein	# 2		Blood pressure Fetal heart rate with Doppler Symphysis fundus height Plasma-glucose Blood tests Urine testing for protein
32	# 4	Blood pressure Fetal heart rate with Doppler Symphysis fundus height			
35	# 5	Blood pressure Fetal heart rate with Doppler Symphysis fundus height Fetal presentation	# 3		Blood pressure Fetal heart rate with Doppler Symphysis fundus height Fetal presentation
37	# 6	Blood pressure Fetal heart rate with Doppler Symphysis fundus height Fetal presentation			
38			# 4		Blood pressure Fetal heart rate with Doppler Symphysis fundus height Fetal presentation
39	# 7	Blood pressure Fetal heart rate with Doppler Symphysis fundus height Fetal presentation			
41	# 8	Blood pressure Fetal heart rate with Doppler Symphysis fundus height Fetal presentation Membrane sweep		# 2	Up-dating history

The information given during pregnancy to the pregnant women is listed in Table 2. In early pregnancy and during the virtual meeting in gestational week 25 most of the information is provided.

**Table 2:** Information given during pregnancy in the standard antenatal care program (SAC) and the modified antenatal care program (MAC)

**Antenatal care program**	SAC/MAC	SAC/MAC	SAC/MAC^a^	SAC/MAC	SAC	SAC	MAC	SAC/MAC
Gestational week	6 to 10	11 to 15	25	29	32	35	35	37/38
Type of information related to pregnancy								
Diet	X		X					
Tobacco	X		X		X		X	
Alcohol	X				X		X	
Medication/Drugs/Violence	X							
Exercise/Sex	X							
Prenatal testing	X							
Social insurance system		X						
Fetal movement			X					
Parent support			X					
Breastfeeding			X					
Family law				X				
Delivery and maternity ward				X				
Contraception								X

*SAC* standard antenatal care program, *MAC* Modified antenatal care program

**Table 3:** Variables included in the composite outcomes and other variables for evaluation of the MAC program

Antenatal care outcomes	Obstetric outcomes	Neonatal outcomes
Question about experience of violence and alcohol habits Recommended weight gain for women with body mass index (BMI) 19.0–29.9 Fear of childbirth-counselling	Premature birth < gestational week 34 + 0 Pregnancy-induced hypertension Preeclampsia Eclampsia	Small for gestational age Large for gestational age Apgar > 7 at 5 min Admission to neonatal intensive care unit
Tobacco use Breastfeeding initiation Breastfeeding 4 weeks postpartum Treatment for mental illness Self-judged health	Gestational diabetes mellitus Anemia Urinary tract infection Pregnancy complications e.g. intrahepatic cholestasis, premature contractions	Intrauterine fetal death Other neonatal complications e.g. jaundice, hypoglycemia
Newborn small for gestational age that has been identified	Undiagnosed fetal breech presentation Induction of labor Vaginal birth after cesarean Instrumental delivery Cesarean section Maternal death

**Table 4:** Direct and Indirect costs to be evaluated in the MAC study

**Healthcare economics:**	
Direct costs:	
outpatient care:	hours spent by midwife
	hours spent by obstetrician
	hours spent by administrators
	hours spent by nurse assistants
	physiotherapy
	psychological treatment
	transportation
inpatient care:	length of stay
cost of implementation:	care level
	Information material Information sessions Education Support from the implementation group during implementation Surveillance Equipment for online communication
Indirect cost:	production loss due to healthcare visits

An in-person visit in the MAC study is defined as an in-office visit between a pregnant woman and a midwife for assessing maternal or fetal well-being. Visits solely for e.g. blood sampling are not counted. For virtual meetings, the video-meeting solution adopted by the included ACCs will be used.

If additional visits are called for during pregnancy, e.g. elevated blood pressure, signs of depression, deviating oral glucose tolerance test, follow-up is individualized. The additional visits could be either back to the midwife for closer surveillance, or booked appointments with other health care staff.

#### Development of questionnaires

Two sets of questionnaires have been developed to assess the pregnant women’s and midwives’ views on quality of care and work situation/condition respectively. The questionnaires are called Patient Reported Experience Measures (PREM 1 and 2) and Midwife Reported Experience Measures (MREM 1 and 2). These questionnaires are seen as Supplements 2, 3, 4, 5 in the original language (Swedish). Some of the questions in PREM are inspired by the PREM questions in the Swedish Pregnancy Register. The questions in MREM also reflect these questions but from a caregiver perspective, including questions important for the implementation evaluation. Both PREM and MREM have two versions. Version 1 is designed to measure the status before the implementation of the MAC and version 2 measure these aspects after the implementation. The MREM is used at two time points after the start of the MAC to get feedback from the midwives and if needed to adjust the implementation strategies and/or the program. To ensure the quality of the questionnaires they are validated face-to-face and revised in an iterative process by an expert panel consisting of experienced researchers, health care professionals and responders similar to the target groups.

#### Development of information to the pregnant women and midwives

Information about the upcoming change of the antenatal care program for healthy pregnant women with a low risk for adverse outcomes is developed and planned to be communicated to the general public with the help of the administration for health communication.

The same unit also develops a communication tool for the midwife to use when communicating with the pregnant women about questions that concern the MAC program.

#### Recruitment of antenatal care centers

Six ACCs in the South East region of Sweden with the same SAC program have been informed about the MAC study and five have agreed to participate. They are called ACC A-E in the study. The participating ACCs have approximately 8200 childbirths annually in total.

#### Randomization

A randomization will be performed for the order in which the ACCs will implement the MAC program. A stepped wedge cluster design was chosen to allow participating centers to serve as control and intervention groups respectively [20]. The intervention represents a change in a clinical routine for all pregnant women in the ACCs with the MAC program, making it unrealistic to randomize by individual.

#### Implementation strategies

In order to achieve a successful implementation, the change to the MAC program in each ACC will follow an implementation plan consisting of a step-by step introduction, with a plan-do-study-act design that offers opportunities to evaluate the implementation and, if necessary, facilitate adherence to the program [21–23]. Implementation strategies involve scheduling dialog meetings between research group and midwives where the research group and the midwives could discuss the need for change, trying to reach a common understanding of “why”, and in later meetings “how” and “when” the change will take place. The midwives will have written instructions for the changed routines, and checklists including information about how to get support, both from the research team and the technology department. Further dialog meetings will be held for feedback, to exchange experiences including short- and long-term evaluation, and to resolve needs for improvement of various kinds during the implementation. Additional dialog meetings will also be held when needed.

#### Participants

All pregnant women will be assessed in the first trimester and classified according to risk, see Supplement 1, and eligibility for the MAC program. This will be done by their midwife, with support when needed from senior colleagues and obstetricians.

In this study the definition of healthy pregnant women with a low risk for adverse outcomes eligible for the MAC program is as follows (see supplement 1):

1. Low risk assessment in four dimensions (psychiatric, social, medical and obstetrical health).
2. Low risk in risk assessment for developing preeclampsia. The assessment for the risk of developing preeclampsia is carried out in the first trimester, based on the uterine pulsatility index, medical history, biochemical markers (PLGF, s-hcg PAPP-A) and median blood pressure in both arms. The pregnant women are classified as either low or high risk for preeclampsia before gestational week 37 and 0 days according to this routine [24].
3. Possibility for the pregnant woman to download an app for virtual meetings and use it.
4. Fluent in Swedish or English

**Fig. 1:**
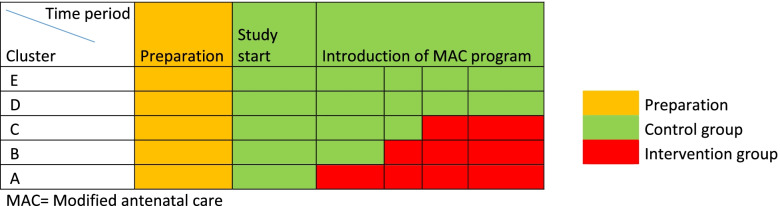
Stepped wedge inclusion. Flow chart of the stepped wedge inclusion of Clusters A-E

On different dates, three of the five ACCs will switch to the MAC program for healthy pregnant women with a low risk for adverse outcomes; the other two will remain as control groups, see Fig. 1.

#### Data collection and timeline

The first step will consist of educational meetings with the midwives at the participating ACCs, with emphasis on information about why this change would be carried out. Three of the five ACCs (A-C) will be included as controls from study start. The two other ACCs (ACC D-E) will begin as control groups three months after the study start. ACC A will be starting with the MAC program four months after the study start followed by ACCs B and ACC C that will begin the MAC program six and seven months respectively after study start. ACCs D-E will remain as control groups. The first data collection will take place approximately 1.5–2 years after study start.

National registers in Sweden offer a possibility to assess the impact of the MAC program on antenatal, obstetric and neonatal outcomes. Healthy pregnant women with a low risk for adverse outcomes will be registered in the Swedish Pregnancy Register in the first trimester as planned to follow either the SAC or the MAC program. Data will also be extracted from medical records in the Obstetrix^©^ and Cosmic^©^ databases as well as local databases.

**Fig. 2:**
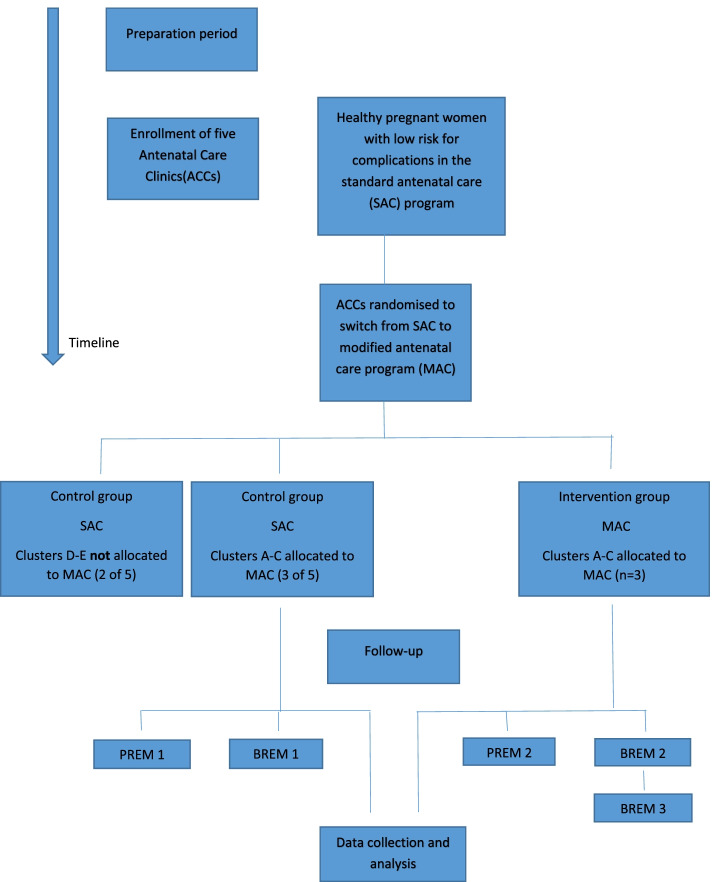
Flowchart. Flowchart of the Modified Antenatal Care program (MAC) study

During the study period, the pregnant women will receive a web survey around gestational week 37–40, with emphasis on their experience of the given care. Healthy pregnant women with a low risk for adverse outcomes in the SAC program (PREM1, Supplement 2) and the MAC program (PREM2, Supplement 3) will receive the survey. The participating midwives will be given a survey before the start of the MAC program (MREM1, Supplement 4), and at two time points after the start of the MAC program (MREM 2, Supplement 5) (Fig. 2).

#### Quality assurance and monitoring of adhesion to the MAC program

The risk assessment protocol (Supplement 1) has been in use for several years in participating ACCs and has been in use by the midwives since 2016. The risk calculation for developing preeclampsia before gestational week 37 and 0 days [24] is also well established, starting the same year as the risk assessment routine. An evaluation of the 150 first risk assessments in early pregnancy will be scrutinized by a research assistant after the start of the MAC program to ensure that the new routine is applied.

### Outcomes

#### Health

This is a non-inferiority trial with the aim of determining if the MAC program differs in quality and safety compared with the SAC program as regards antenatal, obstetric and neonatal outcomes, including satisfaction of the patient and caregiver. The cost-effectiveness and the implementation of the program will also be assessed. Many outcomes measured in this study are rare, since the MAC program only involves a specific low-risk healthy pregnant population. For this reason, some of the outcomes are clustered in the following composites: antenatal care – measuring the national quality measures of the antenatal care program, obstetric- and neonatal outcomes and health care consumption – measuring frequency and setting of visits. The different outcome composites are listed in Tables 3 and 4.

#### Health economic evaluation

Since this study has a non-inferiority design, it is of great interest to conduct a cost-minimization analysis (CMA) [25, 26]. This will make it possible to determine which program reaches a specified health outcome in a given population for the least cost. This kind of analysis also makes it possible to calculate the costs that would arise if every pregnant woman would receive the same care, opening up discussion about redirecting costs toward patient groups that would need more health care. If the results of the MAC program and the SAC program should diverge significantly, a cost-effectiveness analysis will be carried out instead. In that case, the result will be presented as an incremental cost-effectiveness ratio (ICER), where the additional cost, or potential lower cost, will be put in relation to the difference in effect [27].

### Sample size and power calculation

The sample size was calculated with a stepped wedge sample size add-on program to Stata. The program can only detect minimum difference between two groups for superiority designs; therefore, the program was used to calculate the minimum difference that can be detected with a given sample size. It was estimated that a minimum of 1500 pregnant women a year will be possible for the MAC program. The average number of pregnancies per month in the clusters will be approximately 46 and was inserted in the program in ten steps, excluding the baseline. This study population will be enough for our primary outcome, the obstetric composite. The minimum detectable difference with a p-value of 5% and 80% power will be larger than what is estimated in the main non-inferiority hypothesis, resulting in the effect size of 4%. The analysis will be based on the principle of intention to treat.

### Data quality assurance

The answered questionnaires will be checked by research staff for errors and missing data. All cases of intrauterine death will go through an audit and will be monitored specifically by a senior consultant in obstetrics not involved in the study.

The study follows the Standard Protocol Items: Recommendations for Interventional Trials (SPIRIT) statement. All data collected will be stored in the study database in a safe server.

## Ethics

The MAC program will be thoroughly evaluated considering medical safety, but also patient and midwives’ experiences. The Swedish Ethical Review Authority Dr no 2020–03,801 has approved the evaluation of the MAC program.

The pregnant women have the right according to Swedish Law to decline any aspect of care or to change to a different caregiver.

All data that is collected will be coded and results published only on group level. Great efforts will be made to ensure that the women will be given sufficient information regarding the recommended antenatal care program according to their individual risk assessment.

From a fairness perspective, it is crucial to provide care based on individual needs. Swedish health care is obliged to give care on equal conditions for the entire population, but that does not mean the exact same care to everyone. The person that has the greatest need should be given the most care, and the health care system must at the same time strive to be cost-efficient. Hopefully, this study could be useful in that endeavor.

## Trial status

The study preparation period began in 2019 and the first recruitment to the control group started1^st^ of October 2020. The first ACC changing to the MAC program was the 1st of February 2021. The study is ongoing during the whole of 2021–2022 with the first data collection planned for the end of 2022.

## Discussion

Since publicly funded resources are limited, it is important to evaluate whether proper care is given, to ensure patient safety but also to investigate whether unnecessary care is provided or not.

When introducing the MAC program the research team has chosen a high safety approach when defining “healthy pregnant women with a low risk for adverse outcome”. The MAC program could in the future provide a solid base to the antenatal care, and with minor additional services, for example psychological support, to women with fear of childbirth or psychosocial risk factors. This could probably be done virtually as well, but has to be studied more closely. The MAC program could probably also be combined with remote monitoring, e.g. of blood pressure if extra checkups are needed [28].

The stepped wedge cluster design is practical when it is difficult to provide the intervention to all participants at the same time, in this study due to logistical and practical reasons. It is also appropriate for a cost-effectiveness analysis of an intervention on a population basis.

A disadvantage of this study design is the difficulty in blinding the participants. It is also likely to lead to a longer trial duration than a traditional parallel design study. Stepped wedge trials can also be impacted by wider secular trends that could differ between the beginning and the end of the study period. During the preparation period of the MAC study, there was a national change in recommendations for the surveillance of prolonged pregnancy, meaning pregnancy beyond week 41, due to the SWEdish Post Induction Study [29]. This change of routine will be implemented just months before the study start of the MAC study and therefore performed in all study groups. A matter of discussion is how the change of the antenatal care program might affect the spontaneous onset of childbirth. Membrane sweeping has proven to be a valuable tool in trying to lower the induction rates due to prolonged pregnancy [30, 31]. In the SAC program, there is a recommendation for membrane sweeping in pregnancy week 41. In the MAC program on the other hand, there is a membrane sweeping at the prolonged pregnancy check-up at the beginning of pregnancy week 42.The proportion of induction of labor will be evaluated in this study.

Identifying intrauterine growth restriction (IUGR) is one of the most important tasks for midwives at ACCs since a fetus with IUGR has a higher risk of intrauterine fetal death and of being born small for gestational age (SGA). Most of the cases of IUGR are identified due to specific medical guidelines concerning pregnant women with elevated risk for adverse outcomes that recommends extra ultrasounds to assess the fetal growth. Pay et al. stated in their study that the Symphysis-fundus (SF) height can serve as a clinical indicator along with other clinical findings, current and previous medical history, and that it is important to be aware of the limitations of this test, since the SF height has high false negative rates [32]. The risk of missing a fetus with IUGR in the MAC program will be examined in this study through scrutinizing the medical records. The results from the MAC study will be published in scientific papers but also communicated to the heads of antenatal care in all counties in Sweden.

## Supplementary Information

**Additional file 1. **Risk assessment protocol for adverse pregnancy outcomes using four dimensions, in brief summary.

**Additional file 2. **First questionnaire with Patient Reported Experience Measures.

**Additional file 3. **Second questionnaire with Patient Reported Experience Measures.

**Additional file 4. **First questionnaire with Midwife Reported Experience Measures, also called BREM 1. (midwife eng – barnmorska swe).

**Additional file 5. **Second questionnaire with Midwife Reported Experience Measures, also called BREM 2.

## Data Availability

Protocols and questionnaires can be available upon request. Anonymously datasets can upon reasonable request along with appropriate permissions be provided.

## Abbreviations

SAC: Standard Antenatal Care
MAC: Modified Antenatal Care
ACC: Antenatal Care Center
PREM: Patient Reported Experience Measures
MREM: Midwife Reported Experience Measures
IUGR: Intrauterine growth restriction
SF: Symphysis-fundus
