# Supplementary material for: Study protocol for a modified antenatal care program for pregnant women with a low risk for adverse outcomes—a stepped wedge cluster non-inferiority randomized trial
Source: BMC Pregnancy Childbirth. 2022 Apr 8;22:299. doi: 10.1186/s12884-022-04406-7 (PMC8990275; doi:10.1186/s12884-022-04406-7)
Supplement: Supplementary file 1 — Additional file 1. Risk assessment protocol for adverse pregnancy outcomes using four dimensions, in brief summary. [file 12884_2022_4406_MOESM1_ESM.docx]

| Low risk is defined as:  Low risk in all four dimensions, including low risk for preeclampsia. | High risk is defined as:  Risk is identified in any of the four dimensions or  risk for preeclampsia is high. | |
| --- | --- | --- |
| **Psychiatric health**  Low risk   - No previous or ongoing psychiatric illness, or well-monitored/treated minor psychiatric illness   High risk   - Serious previous or ongoing psychiatric illness | | **Social health**  Low risk   - Own accommodation/occupation/economy - Existing social network - No abuse of alcohol or drugs   High risk   - Need for interpreter - Been exposed to violence |
| **Medical health**  Low risk   - Previously healthy, or uncomplicated disease not affecting the pregnancy   High risk   - Body mass Index (BMI )≤ 18 or ≥ 30 kg/m^2^ - Age < 18 or ≥ 40 years. - Intercurrent disease | | **Obstetric health**  Low risk   - Healthy nulliparous or multiparous women with previous normal pregnancy and delivery   High risk   - Oocyte donation - Previous intrauterine fetal death - Previous complicated delivery - Severe fear of childbirth |

Supplement 1. Risk assessment protocol for adverse pregnancy outcomes using four dimensions; Psychiatric- social- medical and obstetric health, in brief summary.
