# Supplementary material for: Study protocol for a modified antenatal care program for pregnant women with a low risk for adverse outcomes—a stepped wedge cluster non-inferiority randomized trial
Source: BMC Pregnancy Childbirth. 2022 Apr 8;22:299. doi: 10.1186/s12884-022-04406-7 (PMC8990275; doi:10.1186/s12884-022-04406-7)
Supplement: Supplementary file 2 — Additional file 2. First questionnaire with Patient Reported Experience Measures. [file 12884_2022_4406_MOESM2_ESM.docx]

Kvinnohälsan


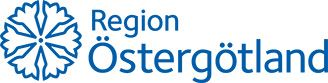


Du har varit på kontroller på mödrahälsovården/ Kvinnohälsan i Östergötland. Vi är intresserade av vad
du tycker om vården.

Vi ber dig därför svara på en enkät om dina erfarenheter. Det är helt frivilligt att svara på enkäten. Svaren
kommer att sammanställas så att inga enskilda individers svar kan identifieras. Det är helheten som är
intressant och svaren kommer att användas för att förbättra verksamheten i framtiden.

Enkäten tar ca 10 minuter att besvara. Kom ihåg att trycka på knappen ” Skicka in ditt svar” i slutet av
enkäten så att dina svar skickas in.

* Obligatorisk fråga

1. I vilken grad har du, utifrån dina behov, kunnat komma i kontakt med
barnmorskan på mödrahälsovården? *

I mycket hög grad

I hög grad

I ganska hög grad

I ganska låg grad

I låg grad

I mycket låg grad

Kan ej/vill ej svara

Inte aktuellt

2. I vilken grad har du kunnat ställa de frågor du önskat till barnmorskan? *

I mycket hög grad

I hög grad

I ganska hög grad

I ganska låg grad

I låg grad

I mycket låg grad

Kan ej/vill ej svara

Inte aktuellt

3. I vilken grad har du fått tillräckligt med information rörande din graviditet
från mödrahälsovården? *

I mycket hög grad

I hög grad

I ganska hög grad

I ganska låg grad

I låg grad

I mycket låg grad

Kan ej/vill ej svara

Inte aktuellt

4. I vilken grad har du känt dig delaktig i planering och beslut som rör din
graviditet i mötet med barnmorskan? *

I mycket hög grad

I hög grad

I ganska hög grad

I ganska låg grad

I låg grad

I mycket låg grad

Kan ej/vill ej svara

Inte aktuellt

5. I vilken grad har du känt dig trygg med barnmorskan på
mödrahälsovården? *

I mycket hög grad

I hög grad

I ganska hög grad

I ganska låg grad

I låg grad

I mycket låg grad

Kan ej/vill ej svara

Inte aktuellt

6. I vilken grad har du känt att du fått stöd av barnmorskan t.ex. om du känt
oro, rädsla, ångest eller motsvarande? *

I mycket hög grad

I hög grad

I ganska hög grad

I ganska låg grad

I låg grad

I mycket låg grad

Kan ej/vill ej svara

Inte aktuellt

7. I vilken grad har du känt att barnmorskan har medverkat till att göra din
partner/närstående delaktig i den utsträckning som du har önskat? *

I mycket hög grad

I hög grad

I ganska hög grad

I ganska låg grad

I låg grad

I mycket låg grad

Kan ej/vill ej svara

Inte aktuellt

8. I vilken grad har du känt dig nöjd med din sammantagna
mödrahälsovård? *

I mycket hög grad

I hög grad

I ganska hög grad

I ganska låg grad

I låg grad

I mycket låg grad

Kan ej/vill ej svara

Inte aktuellt

9. Vilka förväntningar hade du på din mödrahälsovård? *

Övervägande positiva förväntningar

Övervägande negativa förväntningar

Hade inga särskilda förväntningar

Vet inte

10. Hur har dina förväntingar på mödrahälsovården hittills uppfyllts? *

Bättre än förväntat

Samma som förväntat

Sämre än förväntat

11. Hur många besök hos barnmorska har du haft hittills på Kvinnohälsan
under din graviditet? *

1-4 besök

5-9 besök

10 eller fler besök

12. Kan du tänka dig att något/några av besöken skulle kunna ske via
videomöte istället för på plats på Kvinnohälsan, om du mådde bra? *

Ja

Nej

Vet ej

Kommentar till
föregående fråga
angående vide-
omöte?

13. Hur lång är din resväg uppskattningsvis till den mödrahälsovård som du
vanligtvis har besökt? *

km *

14. Vilket färdsätt använder du vanligtvis till din mödrahälsovårdscentral? *

Bil

Buss/tåg

Cykel/går

Annat, vilket?

15. Hur mycket tid har du uppskattningsvis avsatt för att göra ett besök på
mödrahälsovårdscentralen (inklusive restid fram och tillbaka)? *

timme/timmar * minuter *

15. Hur mycket tid har du uppskattningsvis avsatt för att göra ett besök på
mödrahälsovårdscentralen (inklusive restid fram och tillbaka)? *

timme/timmar * minuter *

16. Hur många av dina mödrahälsovårdsbesök genomförde du på betald
arbetstid? *

Alla

Mer än hälften av besöken

Hälften av besöken

Mindre än hälften av besöken

Aldrig

17. Vad är din ålder? *

år *

18. Vilken graviditetsvecka är du i? *

Vecka *

19. Hur många barn har du fött? *

stycken *

20. Vilken är din högsta utbildningsnivå? *

Ingen eller skolgång kortare än 9 år

Grundskola eller motsvarande

Gymnasieskola eller yrkesskola

Högskola/Universitet

21. Om du vill tillägga något, skriv gärna det här!

21. Om du vill tillägga något, skriv gärna det här!
