# Supplementary material for: Study protocol for a modified antenatal care program for pregnant women with a low risk for adverse outcomes—a stepped wedge cluster non-inferiority randomized trial
Source: BMC Pregnancy Childbirth. 2022 Apr 8;22:299. doi: 10.1186/s12884-022-04406-7 (PMC8990275; doi:10.1186/s12884-022-04406-7)
Supplement: Supplementary file 4 — Additional file 4. First questionnaire with Midwife Reported Experience Measures, also called BREM 1. (midwife eng – barnmorska swe). [file 12884_2022_4406_MOESM4_ESM.docx]

[
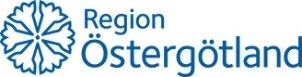
](https://www.regionostergotland.se/contentassets/7163600210ad40aba14bdc3d7ca95639/ro_v_cmyk.eps)

*Enkäten handlar om hur Du upplever Dina möjligheter att tillgodose de gravida kvinnornas olika behov samt om dina förväntningar på nya basprogrammet.*

|  | Vid frågor med svarsalternativ kryssar du i den ruta för det alternativ som stämmer bäst för dig, vid övriga frågor skriver du svaret | | | | | | | | | | | |
| --- | --- | --- | --- | --- | --- | --- | --- | --- | --- | --- | --- | --- |
|  | Fråga 1-7:  I vilken grad har du möjlighet att … | I mycket  hög grad | I hög grad | | I ganska  hög grad | I ganska  låg grad | | I låg grad | I mycket  låg grad | Kan ej/vill ej svara | | Inte aktuellt |
| 1 | vara tillgänglig för de gravida kvinnorna? | 🞎 | 🞎 | | 🞎 | 🞎 | | 🞎 | 🞎 | 🞎 | | 🞎 |
| 2 | svara på de gravida kvinnornas frågor? | 🞎 | 🞎 | | 🞎 | 🞎 | | 🞎 | 🞎 | 🞎 | | 🞎 |
| 3 | ge tillräckligt med information rörande deras graviditet? | 🞎 | 🞎 | | 🞎 | 🞎 | | 🞎 | 🞎 | 🞎 | | 🞎 |
| 4 | göra dem tillräckligt delaktiga i planering och beslut som rörde deras graviditet? | 🞎 | 🞎 | | 🞎 | 🞎 | | 🞎 | 🞎 | 🞎 | | 🞎 |
| 5 | skapa trygghet i mötet med de gravida utifrån det arbetssätt ni har? | 🞎 | 🞎 | | 🞎 | 🞎 | | 🞎 | 🞎 | 🞎 | | 🞎 |
| 6 | ge stöd till de gravida kvinnorna när behov uppstår, t.ex. om de känner oro, rädsla, ångest eller motsvarande? | 🞎 | 🞎 | | 🞎 | 🞎 | | 🞎 | 🞎 | 🞎 | | 🞎 |
| 7 | göra deras partner/närstående delaktiga i ert möte i den utsträckning du uppfattar att de gravida kvinnorna önskar? | 🞎 | 🞎 | | 🞎 | 🞎 | | 🞎 | 🞎 | 🞎 | | 🞎 |
| 8 | I vilken grad känner du dig nöjd med att dina möjligheter/MVHs att tillgodose behoven hos de gravida kvinnorna? | 🞎 | 🞎 | | 🞎 | 🞎 | | 🞎 | 🞎 | 🞎 | | 🞎 |
| 9 | I vilken grad känner du dig nöjd med nuvarande arbetssätt för efterkontroll av förlösta kvinnor? | 🞎 | 🞎 | | 🞎 | 🞎 | | 🞎 | 🞎 | 🞎 | | 🞎 |
| 10 | Vilka förväntningar har du på det nya basprogrammet för friska gravida kvinnor med låg komplikationsrisk? | 1 🞎 Övervägande positiva förväntningar | | | | | | | | | | |
|  |  | 2 🞎 Övervägande negativa förväntningar | | | | | | | | | | |
|  |  | 3 🞎 Har inga särskilda förväntningar | | | | | | | | | | |
|  |  | 4 🞎 Vet inte | | | | | | | | | | |
| 11 | Hur många år har du arbetat som barnmorska | 1 🞎 0 till 2 år | | 2 🞎 från 2 - till 5 år | | | 3 🞎 från 5 -till 10 år | | | | 4 🞎 över 10 år | |
| 12 | Vad är din ålder i år? | …………………………år | | | | | | | | | | |
| 13 | Om du vill tillägga något, skriv gärna det här eller på baksidan! | | | | | | | | | | | |
