# Supplementary material for: Study protocol for a modified antenatal care program for pregnant women with a low risk for adverse outcomes—a stepped wedge cluster non-inferiority randomized trial
Source: BMC Pregnancy Childbirth. 2022 Apr 8;22:299. doi: 10.1186/s12884-022-04406-7 (PMC8990275; doi:10.1186/s12884-022-04406-7)
Supplement: Supplementary file 5 — Additional file 5. Second questionnaire with Midwife Reported Experience Measures, also called BREM 2. [file 12884_2022_4406_MOESM5_ESM.docx]

**BREM 2 Enkäten är frivillig och anonym. Om du inte vill svara på en specifik fråga kan du utelämna denna.** *Enkäten handlar om hur du upplever dina möjligheter att tillgodose de gravida kvinnornas olika behov samt om dina förväntningar på nya basprogrammet* *( basprogram B)*

|  | Vid frågor med svarsalternativ kryssar du i den ruta för det alternativ som stämmer bäst för dig, vid övriga frågor skriver du svaret | | | | | | | | |
| --- | --- | --- | --- | --- | --- | --- | --- | --- | --- |
|  | **Fråga 1-7 gäller ALLA gravida oberoende om de går basprogram A eller B.**  **I vilken grad har du möjlighet att kunna …** | 6  I mycket  hög grad | 5  I hög grad | 4  I ganska  hög grad | 3  I ganska  låg grad | 2  I låg grad | 1  I mycket  låg grad | 9  Kan ej/vill ej svara | 99  Inte aktuellt |
| 1 | vara tillgänglig för de gravida kvinnorna? | 🞎 | 🞎 | 🞎 | 🞎 | 🞎 | 🞎 | 🞎 | 🞎 |
| 2 | svara på de gravida kvinnornas frågor? | 🞎 | 🞎 | 🞎 | 🞎 | 🞎 | 🞎 | 🞎 | 🞎 |
| 3 | ge tillräckligt med information rörande deras graviditet? | 🞎 | 🞎 | 🞎 | 🞎 | 🞎 | 🞎 | 🞎 | 🞎 |
| 4 | göra dem tillräckligt delaktiga i planering och beslut som rörde deras graviditet? | 🞎 | 🞎 | 🞎 | 🞎 | 🞎 | 🞎 | 🞎 | 🞎 |
| 5 | skapa trygghet i mötet med de gravida utifrån det arbetssätt ni har? | 🞎 | 🞎 | 🞎 | 🞎 | 🞎 | 🞎 | 🞎 | 🞎 |
| 6 | ge stöd till de gravida kvinnorna när behov uppstår, t.ex. om de känner oro, rädsla, ångest eller motsvarande? | 🞎 | 🞎 | 🞎 | 🞎 | 🞎 | 🞎 | 🞎 | 🞎 |
| 7 | göra deras partner/närstående delaktiga i ert möte i den utsträckning du uppfattar att de gravida kvinnorna önskar? | 🞎 | 🞎 | 🞎 | 🞎 | 🞎 | 🞎 | 🞎 | 🞎 |
|  | **Fråga 8-11 gäller gravida som går basprogram B** |  |  |  |  |  |  |  |  |
| 8 | I vilken grad känner du dig nöjd med att dina möjligheter att tillgodose behoven hos de friska gravida med låg risk för komplikationer? | 🞎 | 🞎 | 🞎 | 🞎 | 🞎 | 🞎 | 🞎 | 🞎 |
| 9 | I vilken grad känner du dig nöjd med det nya basprogrammet (basprogram B) för de friska gravida med låg risk för komplikationer? | 🞎 | 🞎 | 🞎 | 🞎 | 🞎 | 🞎 | 🞎 | 🞎 |
| 10 | Vilka förväntningar hade du på det nya basprogrammet (basprogram B) för friska gravida med låg risk för komplikationer innan du började arbeta med det? | 1 🞎 Övervägande positiva förväntningar | | | | | | | |
|  |  | 2 🞎 Övervägande negativa förväntningar | | | | | | | |
|  |  | 3 🞎 Hade inga särskilda förväntningar | | | | | | | |
|  |  | 4 🞎 Vet inte → Gå vidare till fråga 12 | | | | | | | |
| 11 | Hur har dina förväntningar på det nya basprogrammet (basprogram B) uppfyllts? | 1 🞎 Övervägande bättre än förväntat | | | | | | | |
|  |  | 2 🞎 Ungefär som förväntat | | | | | | | |
|  |  | 3 🞎 Övervägande sämre än förväntat | | | | | | | |
|  |  | 4 🞎 Vet inte | | | | | | | |
| 12 | Om du vill tillägga något, skriv gärna det här eller på baksidan. | | | | | | | | |

**BREM 3** **Enkäten är frivillig och anonym. Om du inte vill svara på en specifik fråga kan du utelämna denna.** *Enkäten handlar om hur du upplever dina förutsättningar att genomföra det nya basprogrammet (basprogram B)*

|  | Vid frågor med svarsalternativ kryssar du i den ruta för det alternativ som stämmer bäst för dig, vid övriga frågor skriver du svaret | | | | | | | | | | | | |
| --- | --- | --- | --- | --- | --- | --- | --- | --- | --- | --- | --- | --- | --- |
|  | **I vilken grad upplever du att …** | | 6  I mycket  hög grad | 5  I hög grad | | 4  I ganska  hög grad | 3  I ganska  låg grad | 2  I låg grad | | 1  I mycket  låg grad | 9  Kan ej/vill ej svara | | 99  Inte aktuellt |
| 1 | du fått tillräckligt med information om motivet för det nya arbetssättet? | | 🞎 | 🞎 | | 🞎 | 🞎 | 🞎 | | 🞎 | 🞎 | | 🞎 |
| 2 | det är motiverat att arbeta på det nya sättet? | | 🞎 | 🞎 | | 🞎 | 🞎 | 🞎 | | 🞎 | 🞎 | | 🞎 |
| 3 | du får svar på dina frågor om det nya arbetssättet från din arbetsledning? | | 🞎 | 🞎 | | 🞎 | 🞎 | 🞎 | | 🞎 | 🞎 | | 🞎 |
| 4 | du känner stöd för det nya arbetssättet från din arbetsledning? | | 🞎 | 🞎 | | 🞎 | 🞎 | 🞎 | | 🞎 | 🞎 | | 🞎 |
| 5 | det finns en lyhördhet hos ledningen inför dina/barnmorskornas upplevelser och synpunkter? | | 🞎 | 🞎 | | 🞎 | 🞎 | 🞎 | | 🞎 | 🞎 | | 🞎 |
| 6 | du känner stöd från dina kollegor i det nya arbetssättet? | | 🞎 | 🞎 | | 🞎 | 🞎 | 🞎 | | 🞎 | 🞎 | | 🞎 |
| 7 | du fått tillräcklig tid för att ställa om och lära dig det nya arbetssättet? | | 🞎 | 🞎 | | 🞎 | 🞎 | 🞎 | | 🞎 | 🞎 | | 🞎 |
| 8 | din utrustning (dator, program, uppkoppling) har fungerat för att kunna arbeta på distans? | | 🞎 | 🞎 | | 🞎 | 🞎 | 🞎 | | 🞎 | 🞎 | | 🞎 |
| 9 | du vet hur du ska få hjälp om utrustningen inte fungerar? | | 🞎 | 🞎 | | 🞎 | 🞎 | 🞎 | | 🞎 | 🞎 | | 🞎 |
| 10 | det har gått att följa det nya basprogrammet så som det är tänkt med färre fysiska besök för friska gravida med låg risk för komplikationer? | | 🞎 | 🞎 | | 🞎 | 🞎 | 🞎 | | 🞎 | 🞎 | | 🞎 |
| 11 | du är trygg med att dessa friska gravida får färre besök? | | 🞎 | 🞎 | | 🞎 | 🞎 | 🞎 | | 🞎 | 🞎 | | 🞎 |
| 12 | du sammantaget är nöjd med det nya arbetssättet i basprogrammet för friska gravida kvinnor med låg risk för komplikationer? | | 🞎 | 🞎 | | 🞎 | 🞎 | 🞎 | | 🞎 | 🞎 | | 🞎 |
| 14 | Har du upplevt att nya basprogrammet ( B) med färre besök frigjort tid som Kvinnohälsan har använt till annat? | | 1□ Ja-> Gå till fråga 15  2□ Nej. Vänligen beskriv varför……………………………………………………………..Gå till fråga 16 | | | | | | | | | | |
| 15 | Hur har den tiden kunnat användas? (flera svar är möjliga) | | 1□ Till gruppen gravida kvinnor med medel/hög risk  2□ Till preventivmedelsbesök  3□Till GCK mottagningar  4□Till administration, journalarbete etc  5□ Till fortbildning och utvecklingsarbete  6□Ja, till annat. Vänligen beskriv vad…………………………………………………………… | | | | | | | | | | |
| 16 | Har du upplevt att nya basprogrammet ( B) med färre besök frigjort tid som DU har använt till annat? | | 1□ Ja-> Gå till fråga 17  2□ Nej. Vänligen beskriv varför……………………………………………………………..Gå till fråga 18 | | | | | | | | | | |
|  |  | |  | | | | | | | | | | |
| 17 | Hur har den tiden kunnat användas?(flera svar är möjliga) | | 1□ Till gruppen gravida kvinnor med medel/hög risk  2□ Till preventivmedelsbesök  3□Till GCK mottagningar  4□Till att sätta mig in i det nya basprogrammet(B)  5□Till att lära mig tekniken för distansmöten  6□Till administration, journalarbete etc  7□ Till fortbildning och utvecklingsarbete  8□Ja, till annat. Vänligen beskriv vad…………………………………………………………… | | | | | | | | | | |
| 18 | Hur många år har du arbetat som barnmorska | | 1 🞎 0 till 2 år | | 2 🞎 från 2 - till 5 år | | | | 3 🞎 från 5 -till 10 år | | | 4 🞎 över 10 år | |
| 19 | Vad är din ålder i år? | …………………………år | | | | | | | | | | | |
| 20 | Om du vill tillägga något, så skriv gärna det här eller på baksidan. | | | | | | | | | | | | |
